# Thermal spin fluctuations in CoCrFeMnNi high entropy alloy

**DOI:** 10.1038/s41598-018-30732-y

**Published:** 2018-08-15

**Authors:** Zhihua Dong, Stephan Schönecker, Wei Li, Dengfu Chen, Levente Vitos

**Affiliations:** 1 Applied Materials Physics, Department of Materials Science and Engineering, KTH-Royal Institute of Technology, Stockholm, SE 10044 Sweden; 2 College of Materials Science and Engineering, Chongqing University, Chongqing, 400030 P.R. China; 3 Department of Physics and Astronomy, Division of Materials Theory, Uppsala University, Box 516, SE 75121 Uppsala, Sweden; 4 Research Institute for Solid State Physics and Optics, Wigner Research Center for Physics, P.O. Box 49, H-1525 Budapest, Hungary

## Abstract

High entropy alloys based on 3d transition metals display rich and promising magnetic characteristics for various high-technology applications. Understanding their behavior at finite temperature is, however, limited by the incomplete experimental data for single-phase alloys. Here we use first-principles alloy theory to investigate the magnetic structure of polymorphic CoCrFeMnNi in the paramagnetic state by accounting for the longitudinal spin fluctuations (LSFs) as a function of temperature. In both face-centered cubic (fcc) and hexagonal close-packed (hcp) structures, the LSFs induce sizable magnetic moments for Co, Cr and Ni. The impact of LSFs is demonstrated on the phase stability, stacking fault energy and the fcc-hcp interfacial energy. The hcp phase is energetically preferable to the fcc one at cryogenic temperatures, which results in negative stacking fault energy at these conditions. With increasing temperature, the stacking fault energy increases, suppressing the formation of stacking faults and nano-twins. Our predictions are consistent with recent experimental findings.

## Introduction

High entropy alloys (HEAs) are a new class of metallic materials composed of multiple principle elements in equal or near-equal atomic proportions^1,2^. They have been receiving significant research interest owing to their exceptional mechanical and functional properties; see, e.g., refs^3–5^. Among various reported HEAs, the equiatomic CoCrFeMnNi (Cantor alloy)^6^ is a popular and important prototype of HEAs based on 3d transition metals. Within a wide range of temperature below its solidus, it usually forms a chemically disordered solid solution in face-centered cubic (fcc) structure at ambient pressure^6–9^. A unique mechanical characteristic of the Cantor alloy is an enhanced strength-ductility combination with decreasing temperature, while maintaining outstanding fracture toughness at cryogenic temperatures, which was demonstrated to be closely related to the formation of nano-twins and stacking faults in deformation^7,10–15^. Furthermore, due to the irreversible pressure-induced phase transition from the fcc to the hexagonal close-packed (hcp) lattice, the presence of the hcp structure was recently observed at ambient conditions^16–18^. In the CoCrFeNi HEA belonging to the same family as the Cantor alloy, lamellae with hcp structure were also reported at ambient conditions when deforming at cryogenic temperatures below 77 K^19^. These experimental findings are in agreement with the recent theoretical prediction regarding the stability of hcp phase at low temperatures^20^.

In contrast to the intensive investigations of the mechanical properties of the Cantor alloy, understanding the magnetic properties remains very scarce in both the fcc and hcp phases. Among the limited number of works, two magnetic transitions from the disordered paramagnetic (PM) to spin glass, and eventually to the ordered ferromagnetic state were experimentally revealed at 93 and 38 K, respectively, in fcc CoCrFeMnNi^21^. The latter is comparable with the Curie temperature of the alloy predicted from *ab initio* calculations^22,23^, i.e., 20~27 K. These low magnetic transition temperatures indicate that the evolution of its mechanical and functional properties with temperature needs to be considered along with the thermally induced magnetic excitations in the PM state.

First-principles calculations based on density-functional theory (DFT) can provide a sound description of magnetic properties of materials with different crystal structures. However, at finite temperature, a proper account of magnetic excitations, both transversal and longitudinal spin fluctuations (LSFs), is of particular challenge for magnetic transition metals and alloys like the Cantor alloy, owing to the lack of a complete theory for itinerant electron magnetism^24,25^. Starting from the disordered local moment (DLM) theory^26–28^, which approximates a PM state with randomly oriented local magnetic moments within the mean-field approximation, several *ab initio* methodologies have been recently proposed to properly describe the LSFs in the PM state^29–33^. The crucial role of LSFs in the accurate description of finite-temperature magnetic properties has been demonstrated in a few magnetic metals including PM bcc (body-centered cubic) and fcc Fe, fcc Ni and fcc Co^30–34^, and in alloys such as PM fcc/hcp Fe-Mn and Fe-Cr-Ni systems^29,35–38^. It was reported that LSFs usually lead to sizable impacts on the temperature-dependent mechanical and physical properties of the metals and alloys, such as single-crystal elastic constants^29,30,35^, lattice expansion^34^ and intrinsic energy barriers associated with fundamental plasticity mechanisms^36–39^. However, to our best knowledge, there has been no attempt yet to explore the role of thermal LSFs in PM CoCrFeMnNi HEA for neither the fcc nor hcp phase, in spite of its high fundamental and practical interest.

In this report, we put forward the first comprehensive description of the magnetic state of PM CoCrFeMnNi HEA in both the fcc and hcp phases by accounting for the LSFs as a function of temperature. The influence of LSFs on the phase stability, stacking fault energy and the fcc-hcp interfacial energy of the alloy are elaborated at finite temperature.

## Results and Discussion

**Figure 1:**
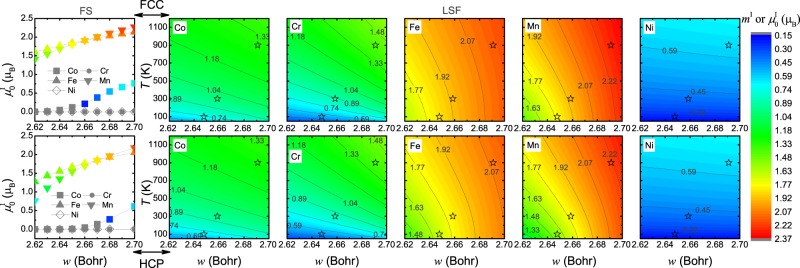
Magnetic moments of alloy components in PM CoCrFeMnNi for the fcc (upper panels) and hcp (lower panels) structure. The first column shows the equilibrium magnetic moments {${\mu }_{0}^{{\rm{I}}}$} (I = Co, Cr, Fe, or Ni) as a function of volume obtained from the conventional FS calculations. The contour plots show the mean magnetic moments {*m*^I^} for the alloy components as a function of volume and temperature when accounting for the LSFs. The experimental volumes^40^ at 100, 300 and 900 K are indicated by stars.

The contour plots in Fig. 1 display the mean magnetic moments {*m*^I^} (I = Co, Cr, Fe, or Ni) of alloy components in PM CoCrFeMnNi as a function of volume (represented by the Wigner-Seitz radius *w*) and temperature *T* for the fcc (upper panels) and hcp (lower panels) structures when considering the effect of LSFs. For comparison, the equilibrium magnetic moments $\{{\mu }_{0}^{{\rm{I}}}\}$ derived from the conventional floating spin (FS) calculations are presented in the left panels, which are solely volume-dependent emerging from the magneto-volume coupling.

For the FS calculations shown in the first column of Fig. 1, a somewhat similar magneto-volume coupling is observed in the fcc and hcp phases for the same alloy component. In particular, in both phases large spontaneous magnetic moments are obtained for Fe and Mn at all considered volumes, whereas those of Cr and Ni are always zero and a finite magnetic moment arises only at large volumes for Co. Comparing the magnitude of $\{{\mu }_{0}^{{\rm{I}}}\}$ in the two phases, Fe, Mn, and Co in the fcc phase exhibit larger magnetic moments than in the hcp phase at the given volumes. The maximum difference amounts to ~0.32, 0.64 and 0.28 *μ*_B_ for Fe, Mn and Co at the radius of ~2.62, 2.62 and 2.68 Bohr, respectively.

Accounting for the LSFs at finite temperature (contour plots in Fig. 1), finite magnetic moments have been thermally induced for Cr, Ni and Co at all volumes even at the lowest temperature considered here (50 K) in both the fcc and hcp phases. The mean magnetic moment *m*^I^ in both phases exhibits a monotonic dependency on volume and temperature. The *m*^I^ of Fe and Mn, the two elements exhibiting large spontaneous static magnetic moments ${\mu }_{0}^{{\rm{I}}}$, shows more pronounced dependency on volume with respect to that on temperature, whereas LSFs give the prevailing contribution to the *m*^I^ of Co, Cr and Ni in both the fcc and hcp phases. As the volume increases, the impact of LSFs on *m*^I^ weakens for all alloy components.

Taking the experimentally determined thermal lattice expansion^40^ into account (indicated by stars in the contour plots in Fig. 1), the mean magnetic moments {*m*^I^} as function of temperature are shown in Table 1 for both the fcc and hcp phases. It is evident that {*m*^I^} in both phases significantly increase with temperature, and at the given temperatures the magnetic states of the alloy components in the fcc phase are very close to those in the hcp phase. At 100 K, the difference in *m*^I^ between the two phases amounts to ~0.05, 0.03, 0.17 and 0.23 *μ*_B_ for Co, Cr, Fe, and Mn, respectively, and this difference slightly reduces with increasing temperature. Furthermore, *m*^Ni^ in the two phases is almost identical at the considered temperatures with difference less than 0.005 *μ*_B_.

**Table 1:** The mean magnetic moments {*m*^I^} of alloy components in PM CoCrFeMnNi as a function of temperature for the fcc and hcp phases when considering the effect of LSFs in combination with thermal lattice expansion.

*T*	fcc					hcp				
	Co	Cr	Fe	Mn	Ni	Co	Cr	Fe	Mn	Ni
100	0.73	0.52	1.85	1.79	0.24	0.67	0.49	1.69	1.57	0.24
300	0.98	0.88	1.94	1.94	0.40	0.94	0.84	1.82	1.80	0.40
900	1.30	1.42	2.15	2.27	0.63	1.27	1.39	2.09	2.21	0.63

*m*^I^ (with I = Co, Cr, Fe, Mn, or Ni) is given in units of *μ*_B_, *T* is in K.

In the following, we elaborate on the influence of LSFs in PM CoCrFeMnNi in the sequence of the lattice stability, the intrinsic stacking fault energy *γ*_*isf*_, and the interfacial energy *σ* between the fcc and hcp phases.

**Figure 2:**
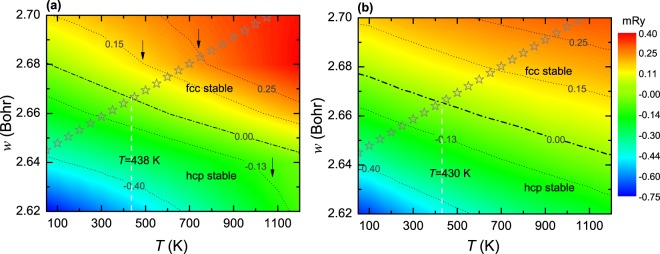
Free energy difference between the fcc and hcp phases, Δ*F* ^fcc→hcp^ = *F* ^hcp^ − *F* ^fcc^, as a function of volume and temperature. For comparison, panel (a and b) show the results derived from the FS and LSF schemes, respectively. The thermal lattice expansion measured in ref.^40^ is indicated by stars.

The free energy differences between the fcc and hcp phases, i.e., Δ*F* ^fcc→hcp^ = *F* ^hcp^ − *F* ^fcc^, derived from the FS and LSF schemes are compared in Fig. 2. Both the FS and LSF results indicate that the lattice stability of PM CoCrFeMnNi noticeably changes with temperature and volume. The hcp phase is thermodynamically stable against the fcc one at low temperatures and small volumes. When the Wigner-Seitz radius is below (above) ~2.64 Bohr (2.68 Bohr), the hcp (fcc) phase is energetically preferable at all considered temperatures ranging from 50 to 1200 K.

**Figure 3:**
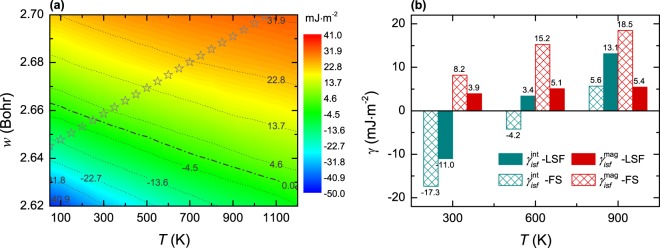
The contour in panel (a) plots for the stacking fault energy *γ*_*isf*_ as a function of volume and temperature when accounting for LSFs. The thermal lattice expansion measured in ref.^40^ is indicated by stars. The bars in panel (b) display the partial contributions to *γ*_*isf*_ from the internal energy ${\gamma }_{isf}^{{\rm{int}}}$ and the magnetic entropy ${\gamma }_{isf}^{{\rm{mag}}}$, i.e., ${\gamma }_{isf}={\gamma }_{isf}^{{\rm{int}}}+{\gamma }_{isf}^{{\rm{mag}}}+2\sigma$, at the given temperatures for the LSF and FS calculations.

With respect to the Δ*F* ^fcc→hcp^ derived from the FS calculations, the LSFs at finite temperature systemically lower the absolute magnitude of Δ*F* ^fcc→hcp^, and promote rather smooth dependencies of Δ*F* ^fcc→hcp^ on volume and temperature, as indicated by straight contour lines. As detailed in Fig. 1, the noticeable changes in the shape of contour lines in the FS calculations [indicated by the arrows in panel (a)] emerge from the distinct {${\mu }_{0}^{{\rm{I}}}$} in the fcc and hcp phases, amplified by temperature in the calculation of magnetic entropy. These changes are significantly eliminated by the gradually varying {*m*^I^} induced via LSFs in both the fcc and hcp phases. That is, the partial contributions to Δ*F* ^fcc→hcp^, i.e., the magnetic entropy and internal energy, may be considerably altered by LSFs at finite temperature (see Fig. 3). Nevertheless, LSFs yield small influence on the phase boundary (defined here as Δ*F* ^fcc→hcp^ = 0) of PM CoCrFeMnNi. Referring to the experimental volumes^40^ indicated by stars, the critical temperature of fcc-hcp phase transition at ambient pressure is determined to be ~430 K in the LSF calculations, compared to ~438 K in the FS calculations. The critical temperature predicted here is in good agreement with the experimental value of ~633 K, especially when taking into account the error bars stated in the experiments^16^ and also the fact that the explicit phonon effect is not considered in the present theory.

Considering an intrinsic stacking fault in fcc alloys as a two-layer embryo in the hcp structure embedded in the fcc matrix, the thermodynamic model proposed by Olson and Cohen^41^ was formulated to evaluate *γ*_*isf*_ in terms of the Δ*F* ^fcc→hcp^ and the interfacial energy *σ* between the two phases. Taking the computed Δ*F* ^fcc→hcp^ and a constant *σ* of 5 mJ m^−2^ (discussed below) as inputs to the model, we calculated the *γ*_*isf*_ of PM CoCrFeMnNi as a function of volume and temperature. The *γ*_*isf*_ derived from the LSF scheme is shown in Fig. 3(a), while the two partial contributions from the internal energy and magnetic entropy, i.e., ${\gamma }_{isf}^{{\rm{int}}}$ and ${\gamma }_{isf}^{{\rm{mag}}}$, respectively, are compared in Fig. 3(b) for the LSF and FS calculations.

It is evident that *γ*_*isf*_ monotonically increases with volume and temperature, showing a tendency to saturate at high temperatures and large volumes. Compared to the FS results (not shown), *γ*_*isf*_ accounting for LSFs decreases in the most fcc-stable region by an upper change of ~13.5 mJ m^−2^ (at 2.68 Bohr and 1200 K), whereas it increases in the most hcp-stable region by below ~6.2 mJ m^−2^ (at 2.62 Bohr and 300 K). Taking the experimentally determined lattice expansion (indicated by the stars) into account, the *γ*_*isf*_ of PM CoCrFeMnNi is predicted from the LSF calculations to increase from −17 mJ m^−2^ at 50 K, reach zero at ~260 K and keep rising to 31 mJ m^−2^ at 1000 K, which are contrasted with −19 mJ m^−2^, ~290 K and 36 mJ m^−2^, respectively, from the FS calculations. The positive temperature dependence of *γ*_*isf*_ predicted here is comparable with that reported by Huang *et al*.^42^. Nevertheless, the magnitudes of *γ*_*isf*_ accounting for LSFs are lower than those found in ref.^42^, because the magnetic contribution determined in the work is significantly reduced owing to LSFs.

Looking at the partial contributions to *γ*_*isf*_ shown in Fig. 3(b), it is evident that LSFs considerably reduce the contribution emerging from the magnetic entropy ${\gamma }_{isf}^{{\rm{mag}}}$ (owing to the similar magnetic states in the fcc and hcp phases, see Fig. 1), whilst noticeably increasing the internal energy part ${\gamma }_{isf}^{{\rm{int}}}$. The positive temperature dependence of *γ*_*isf*_ is ultimately dominated by the magneto-volume coupling at thermal excitations. The role of LSFs disclosed in PM CoCrFeMnNi is in good agreement with that reported in PM *γ*-Fe^39^ and Fe-22.5 at.% Mn^36^.

In the temperature interval 77–293 K nano-twins and stacking faults were reported in experiments^7,10–15^, the *γ*_*isf*_ of PM fcc CoCrFeMnNi is predicted to be as low as −15~2 mJ m^−2^ when accounting for LSFs and lattice expansion. The low *γ*_*isf*_ is attributed to the fact that the hcp phase is thermodynamically stable to the fcc one at these conditions (see Fig. 2). Namely, at cryogenic temperatures the fcc phase would remain metastable, because of, e.g., high kinetic barriers^16,43^, and formation of nano-twins and stacking faults therein is energetically preferable. With decreasing temperature, the plastic deformation by nano-twins and stacking faults is enhanced owing to the reduced *γ*_*isf*_, resulting in a good combination of strength and ductility at cryogenic temperatures as observed in the experiments.

**Figure 4:**
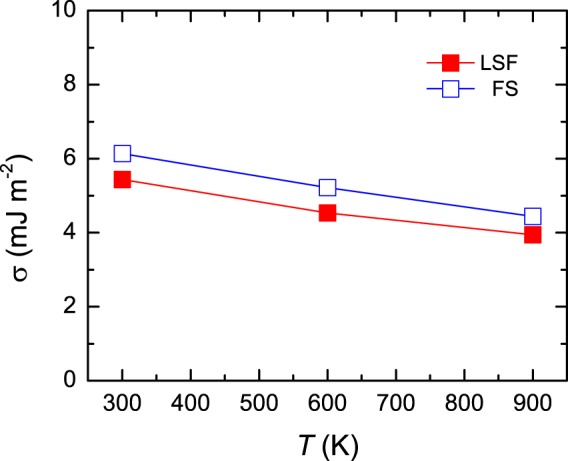
The fcc-hcp interfacial energy *σ* as a function of temperature for PM CoCrFeMnNi. For comparison, *σ* derived from the LSF and FS schemes are presented. The thermal lattice expansion was accounted for using the experimental volumes reported in ref.^40^.

We end our discussion by elaborating on the influence of LSFs on the fcc-hcp interfacial energy *σ*. By using the Δ*F* ^fcc→hcp^ and the *γ*_*isf*_ computed adopting the supercell approach, we calculated *σ* via the thermodynamic model proposed by Olson and Cohen^41^. The *σ* of PM CoCrFeMnNi derived from the LSF and FS schemes is compared in Fig. 4 for different temperatures, where the experimentally determined lattice expansion^40^ was accounted for. It is evident from Fig. 4 that the LSFs at finite temperature slightly lower *σ* by 0.5~0.7 mJ m^−2^ in the temperature interval 300–900 K. As the temperature increases, the *σ* accounting for LSFs slightly decreases at a coefficient of ~0.0025 mJ m^−2^ K^−1^, which is very close to the one derived from the FS calculations, i.e., ~0.0028 mJ m^−2^ K^−1^. Extrapolating our calculations to 0 K, the *σ* of PM CoCrFeMnNi is predicted to be ~6.1 and 7.0 mJ m^−2^ in the LSF and FS schemes, respectively, showing a good consistency with the values calculated for Fe-Cr-Ni austenitic stainless steels by Li *et al*.^44^, i.e., 7.5–9.0 mJ m^−2^ at 0 K.

In the temperature interval 50–1200 K, the *σ* of PM CoCrFeMnNi is estimated to slightly vary in the range of ~3.1–6.0 and 3.6–6.8 mJ m^−2^ for the LSF and FS calculations, respectively. Therefore, the error bar of *γ*_*isf*_ associated with using the constant *σ* equal to 5 mJ m^−2^ is expected to be less than 4 mJ m^−2^ for both the LSF and FS calculations.

## Conclusions

Using *ab initio* alloy theory, we investigated the magnetic structure of PM CoCrFeMnNi HEA in both the fcc and hcp crystal structures by accounting for the LSFs as a function of temperature and volume, and assessed the LSF contributions to finite-temperature properties of the alloy. We show that the LSFs induce sizable magnetic moments for Co, Cr and Ni, whilst slightly increasing the magnetic moment of Fe and Mn in both the fcc and hcp phases. LSFs are demonstrated to give limited influence on the fcc-hcp phase stability, stacking fault energy and the interfacial energy in PM CoCrFeMnNi, but significantly alter the partial contributions of the finite-temperature properties. The hcp phase is energetically preferable against the fcc one at low temperatures and volumes, which is responsible for the negative stacking fault energy at these conditions. Dominated by the magneto-volume coupling at thermal excitations, the stacking fault energy increases with temperature, suppressing the formation of nano-twins and stacking faults. The present predictions are consistent with the recent experimental findings.

## Methods

The LSF methodology proposed by Dong *et al*.^29,30^ was adopted to describe the finite-temperature magnetic state of each alloy species in PM CoCrFeMnNi HEA. The root-mean-square magnetic moment *m*^I^(*w*, *T*)^30^, which is formulated as ${m}^{{\rm{I}}}=\sqrt{\int \,{\mu }^{2}\cdot x(\mu )d\mu }$ with *x*(*μ*) being the spin-density distribution of longitudinal magnetic component *μ*, was used to represent the LSF energetics at thermal excitations in both the fcc and hcp phases. For the sake of computational feasibility of determining {*m*^I^}, we used the ‘fluctuating medium approximation’ and the ‘one shot from static equilibrium approach’^29^. The present LSF methodology has been applied to PM bcc and fcc Fe and Fe-Cr-Ni alloy, and the predicted finite-temperature properties such as single-crystal elastic constants^29,30^, lattice expansion^34^ and intrinsic energy barriers^39^ confirm the accurate description of the magnetic state at elevated temperatures.

The finite-temperature properties were derived from the free energy *F*^*α*^ (*α* represents the fcc or hcp phase) by accounting for lattice expansion and LSFs at finite temperature, which is expressed as *F*^*α*^(*w*, *T*) = *E*^int^(*w*, {*M*^I^}) − *TS*^mag^({*M*^I^}) (all three terms here depend on *α*, but the notation at the right-hand side is omitted for simplicity). While the internal energy *E*^int^ is approximated by the total energy of a DLM paramagnet with local magnetic moments {*M*^I^}, the magnetic entropy *S*^mag^ is evaluated in the mean-field expression via ${S}^{{\rm{mag}}}={k}_{{\rm{B}}}\,{\sum }_{{\rm{I}}}\,{c}^{{\rm{I}}}\cdot \,\mathrm{ln}({M}^{{\rm{I}}}+1)$ (*k*_B_ is the Boltzmann constant and *c*^I^ is the chemical concentration). *M*^I^ represents the equilibrium magnetic moment ${\mu }_{0}^{{\rm{I}}}$ in the conventional FS calculations, while it is the mean magnetic moment *m*^I^ in the LSF calculations. The fcc-hcp phase stability was evaluated by the free energy difference between the two phases, i.e., Δ*F* ^fcc→hcp^ = *F* ^hcp^ − *F* ^fcc^, which also entered the thermodynamic model proposed by Olson and Cohen^41^ to compute the stacking fault energy *γ*_*isf*_. The fcc-hcp interfacial energy *σ* was determined by comparing the results from the thermodynamic model^41^ with the supercell calculations via $2\sigma ={\gamma }_{isf}^{{\rm{SC}}}-2{\rm{\Delta }}{F}^{{\rm{fcc}}\to {\rm{hcp}}}/A$ (*A* is the interfacial area per atom). ${\gamma }_{isf}^{{\rm{SC}}}$ is the stacking fault energy calculated using the supercell approach, in which we assumed that the magnetic state of the atomic layers nearest to the intrinsic stacking fault is the same as in the hcp structure, while the remaining layers were treated identically to the fcc case^39^. Details about the adopted supercell structure can be found in ref.^39^. In the present application, an ideal *c*/*a* ratio of $\sqrt{\mathrm{8/3}}$ was employed for the hcp structure, the finite-temperature volume of which was assumed to be identical with the fcc one. The experimental volumes of fcc CoCrFeMnNi at various temperatures were determined from the measurements reported in ref.^40^ by assuming a linear thermal expansion in the considered temperature interval. The structural relaxation in the faulted supercell was omitted. The *k*-meshes and supercell size were carefully tested to ensure sufficient numerical accuracy.

For the DFT calculations, the Kohn-Sham equations were solved within the framework of the exact muffin-tin orbitals (EMTO) method^45–48^ adopting the scalar-relativistic approximation in combination with the soft-core scheme. The self-consistent electronic structure calculations and the total energy calculations were carried out within the generalized gradient approximation as parametrized by Perdew-Burke-Ernzerhof (PBE)^49^. The magnetic and chemical disorders were described by the DLM picture^26–28^ in combination with the coherent-potential approximation (CPA)^50,51^.
